# Supplementary material for: Læmple: a benchmarking framework for virus lineage deconvolution tools for SARS-CoV-2 from wastewater
Source: GigaByte. 2026 Oct 13;2026:gigabyte193. doi: 10.46471/gigabyte.193 (PMC13688667; doi:10.46471/gigabyte.193)
Supplement: Peer review history

# Reviewer report: Review MS


Reviewer name and names of any other individual's who aided in reviewerLiuyang ZHAODo you understand and agree to our policy of having open and named reviews, and having your review included with the published manuscript. (If no, please inform the editor that you cannot review this manuscript.)YesIs the language of sufficient quality?YesPlease add additional comments on language quality to clarify if neededIs there a clear statement of need explaining what problems the software is designed to solve and who the target audience is? NoAdditional CommentsIs the source code available, and has an appropriate Open Source Initiative license <a href="https://opensource.org/licenses" target="_blank">(https://opensource.org/licenses)</a> been assigned to the code?YesAdditional CommentsAs Open Source Software are there guidelines on how to contribute, report issues or seek support on the code?YesAdditional CommentsIs the code executable?Unable to testAdditional CommentsIs installation/deployment sufficiently outlined in the paper and documentation, and does it proceed as outlined?NoAdditional CommentsIs the documentation provided clear and user friendly?YesAdditional CommentsAdditional CommentsIs there a clearly-stated list of dependencies, and is the core functionality of the software documented to a satisfactory level?YesAdditional CommentsHave any claims of performance been sufficiently tested and compared to other commonly-used packages? NoAdditional CommentsIs test data available, either included with the submission or openly available via cited third party sources (e.g. accession numbers, data DOIs)?YesAdditional CommentsAre there (ideally real world) examples demonstrating use of the software? NoAdditional CommentsAdditional CommentsAny Additional Overall Comments to the AuthorThis paper presents Læmple, a Snakemake-based benchmarking framework for testing SARS-CoV-2 lineage deconvolution tools on wastewater sequencing data. It uses simulated data (via SWAMPy), runs mutation calling, executes a bunch of tools, and spits out standardized performance metrics. The authors test seven tools on three simulated datasets with different complexity levels and seven coverage depths. The main contribution is the framework itself, not a fixed ranking of tools—which they do a decent job of clarifying. Overall, it’s a useful and timely piece for the wastewater surveillance crowd, but there are a number of methodological and presentational issues that need fixing before I’d recommend publication. Major Concerns 1. Simulation only covers Illumina MiSeq data, which is increasingly less representative of real wastewater sequencing workflows. The entire benchmarking relies on simulated data that mimics Illumina MiSeq runs (presumably starting from something like “short, high‑quality reads with low error rates”). But many wastewater surveillance labs have moved to other platforms—Oxford Nanopore (for its low upfront cost, rapid turnaround, and long reads that help with lineage resolution in complex mixtures) or higher‑throughput Illumina instruments like NovaSeq (for deep, cost‑effective population sequencing). MiSeq is fine for some contexts, but it’s not the only game in town anymore, and the error profiles, read length distributions, and coverage biases differ substantially between platforms. 2. No real-world validation at all. Everything is based on simulated data. They never test against actual wastewater samples with known lineage composition. Yes, they say simulation has advantages over spike-ins, but they still have to validate that their simulation actually looks like real data. Even a simple qualitative comparison (coverage uniformity, allele frequency spectra) between simulated and real samples would go a long way. Without that, I’m left wondering whether these benchmark results mean anything in practice. Recommendation: Add a supplementary figure comparing key quality metrics (coverage uniformity, dropout rates, error profiles) between simulated data and real wastewater data. Or just run a small subset of tools on a real dataset with known composition and see if the performance estimates hold up. 3. Lineage nomenclature heterogeneity is not handled well. Different tools use totally different reference sets and lineage granularities—VirPool has 6 variants, QuaID has 3,686 lineages. This fundamentally skews every metric. A tool with 6 reference lineages cannot possibly achieve the same true positive rate as one with thousands, and its precision is measured against a completely different denominator. They sort of acknowledge this for QuaID (WHO-level vs. PANGO-level reporting), but the broader confounding issue isn’t addressed. And their fix for QuaID—splitting WHO-level estimates evenly across constituent PANGO lineages—is completely ad hoc and could distort both PPV and TPR. Recommendation: Provide a clear table of reference set characteristics for each tool. Discuss reference set size as a confound explicitly. Consider reporting metrics at a harmonized taxonomic level (e.g., collapsing to parent lineages) as a sensitivity analysis. Minor Concerns 1. Writing quality needs work. Lots of typos, grammatical errors. 2. No runtime or resource benchmarks. For a practical benchmarking framework, users care about computational cost (runtime, memory, disk). Not reported. 3.Software versioning is a problem. Several tools were installed in 2023 with reference sets from that era. The manuscript appears to be submitted in 2025. The landscape has changed a lot. This shows the value of the framework for longitudinal tracking, but it also means the specific comparative results are likely out of date—needs explicit acknowledgment.RecommendationMajor Revisions


# Reviewer report: Review MS


Reviewer name and names of any other individual's who aided in reviewerZhencheng FangDo you understand and agree to our policy of having open and named reviews, and having your review included with the published manuscript. (If no, please inform the editor that you cannot review this manuscript.)YesIs the language of sufficient quality?YesPlease add additional comments on language quality to clarify if neededIs there a clear statement of need explaining what problems the software is designed to solve and who the target audience is? YesAdditional CommentsIs the source code available, and has an appropriate Open Source Initiative license <a href="https://opensource.org/licenses" target="_blank">(https://opensource.org/licenses)</a> been assigned to the code?YesAdditional CommentsAs Open Source Software are there guidelines on how to contribute, report issues or seek support on the code?YesAdditional CommentsIs the code executable?Unable to testAdditional CommentsIs installation/deployment sufficiently outlined in the paper and documentation, and does it proceed as outlined?Unable to testAdditional CommentsIs the documentation provided clear and user friendly?NoAdditional CommentsIs there enough clear information in the documentation to install, run and test this tool, including information on where to seek help if required?NoAdditional CommentsIs there a clearly-stated list of dependencies, and is the core functionality of the software documented to a satisfactory level?YesAdditional CommentsHave any claims of performance been sufficiently tested and compared to other commonly-used packages? YesAdditional CommentsIs test data available, either included with the submission or openly available via cited third party sources (e.g. accession numbers, data DOIs)?YesAdditional CommentsAre there (ideally real world) examples demonstrating use of the software? NoAdditional CommentsIs automated testing used or are there manual steps described so that the functionality of the software can be verified?YesAdditional CommentsAny Additional Overall Comments to the AuthorThe manuscript emphasizes Læmple as a modular and extensible benchmarking framework. However, the current GitHub documentation remains rather high-level for users who wish to customize the workflow beyond the provided examples. I suggest that the authors expand the documentation with: (i) an annotated configuration file explaining key parameters and providing concrete examples for customizing simulated lineage compositions, time courses, sequencing quality, sampling schemes, reference genomes and primer schemes; (ii) a small worked example showing the expected output directory and explaining how to interpret the key output files, columns, and summary metrics; (iii) a step-by-step tutorial or template for integrating a newly developed deconvolution tool that is not currently included in the workflow, including the required Snakemake subworkflow, conda environment, wrapper script, and standardized output format; and (iv) clearer instructions or a worked example for adapting the framework to viral pathogens other than SARS-CoV-2. This last point is particularly important because the manuscript states that the framework can be easily adapted to other viral pathogens by changing the configuration of the initial workflow module, but the current repository mainly documents SARS-CoV-2-specific examples and resources.RecommendationMajor Revisions


# Reviewer report: Re-Review MS


Indicate in the comments box below whether you are happy with the changes made or if the manuscript is unacceptable.Comments on revised manuscriptThe authors have done their most to enhance the manuscript. As the paper has reached its maximum potential and no further actionable improvements remain, I recommend it for acceptance. Thanks.Indicate in the comments box below whether you are happy with the changes made or if the manuscript is unacceptable.Comments on revised manuscriptThe authors have done their most to enhance the manuscript. As the paper has reached its maximum potential and no further actionable improvements remain, I recommend it for acceptance. Thanks.


# Reviewer report: Re-Review MS


Indicate in the comments box below whether you are happy with the changes made or if the manuscript is unacceptable.Comments on revised manuscriptThe author has addressed my concerns, and I believe the paper is suitable for publication.Indicate in the comments box below whether you are happy with the changes made or if the manuscript is unacceptable.Comments on revised manuscriptThe author has addressed my concerns, and I believe the paper is suitable for publication.


# Editor report: Accept


Editor’s AssessmentThe manuscript is ready for formal acceptance.Editor’s AssessmentThe manuscript is ready for formal acceptance.
